# Barriers and recommendations for a cervical cancer screening program among women in low-resource settings in Lagos Nigeria: a qualitative study

**DOI:** 10.1186/s12889-022-14314-2

**Published:** 2022-10-12

**Authors:** Tope Olubodun, Mobolanle Rasheedat Balogun, Abimbola Kofoworola Odeyemi, Oluwakemi Ololade Odukoya, Adedoyin Oyeyimika Ogunyemi, Oluchi Joan Kanma-Okafor, Ifeoma Peace Okafor, Ayodeji Bamidele Olubodun, Oluwatoyin Olanrewaju Progress Ogundele, Babatunde Ogunnowo, Akin Osibogun

**Affiliations:** 1 Department of Community Health and Primary Care, Lagos University Teaching Hospital, Surulere, Lagos, Nigeria; 2 Olabisi Onabanjo University Teaching Hospital, Sagamu, Ogun State Nigeria; 3 Ladiya hospital. Sabon Gari, Zaria, Kaduna State Nigeria

**Keywords:** Cervical cancer screening, Barriers, Pap smear, Recommendations, Slum, Low-resource settings, Qualitative

## Abstract

**Background:**

Cervical cancer is the fourth most common cancer in women globally despite being a largely treatable and preventable malignancy. Developing countries account for over 80% of all new cases. Women residing in low-resource settings such as those residing in slums have a higher risk of cervical cancer, and lower uptake of cervical cancer screening. Diverse barriers influence the uptake of cervical cancer screening among women in low-resource settings.

**Objectives:**

This qualitative study was done prior to the introduction of a cervical cancer screening program in two slum areas in Lagos Nigeria and explored women’s knowledge about cervical cancer, and their perceived barriers and recommendations for the program.

**Method:**

Four focus group discussions(FGD) were conducted among 35 women between the ages of 21–65 years residing in two urban slums in Lagos, Nigeria from February to April 2019. Each FGD was limited to 8–10 participants of women of similar ages. Voice recordings were transcribed verbatim and thematic analysis was done.

**Results:**

Most of the women were not aware of cervical cancer and none knew the symptoms or risk factors of cervical cancer. The participants felt that the cervical cancer screening program would be well accepted in the community, however, expressed concerns about the cost of the screening test and the sex of the person performing the test. The recommendations proffered for a successful cervical cancer screening program include; reducing the cost of the test or providing the test free of charge, having people that speak the local language as part of the team, using female health care providers, using a private location within the community or nearby primary health center, and publicizing the program with the use of SMS, phone calls, town crier, and health talks. It was recommended that organizing health education sessions would help improve women’s poorly perceived susceptibility to cervical cancer.

**Conclusion:**

Interventions to increase uptake of cervical cancer screening among women in low resource settings need to improve knowledge of cervical cancer and address barriers to cervical cancer screening such as cost, distance, and as much as possible, sex of the healthcare provider should be considered.

**Supplementary Information:**

The online version contains supplementary material available at 10.1186/s12889-022-14314-2.

## Introduction

Worldwide, cervical cancer is the fourth most common cancer in women. Approximately 570,000 cases of cervical cancer and 311,000 deaths from the disease occurred in 2018 [1]. In developing countries cervical cancer remains the second most common cancer in women [2] Cervical cancer incidence and mortality highlight the great disparities that exist between developed and developing countries [3]. Analysis of data from the Global Cancer Observatory 2018 database showed that the age-standardized incidence rate (ASIR) and age-standardized mortality rate (ASMR) of countries in the very high human development index (HDI) tier were 9.6 per 100 000 women and 3.0 per 100 000 respectively, while in countries in the low HDI tier, ASIR was 26.7 per 100 000 and ASMR, 20.0 per 100 000 [4]. High-income countries have experienced a steady decline in incidence and mortality from cervical cancer, which is attributed to well-organized screening programs and infrastructure that provide appropriate follow-up and treatment [3].

According to the Global Strategy for cervical cancer elimination adopted in 2020 by the World Health Assembly, every country should meet the 90-70-90 targets by 2030 i.e. “90% of girls fully vaccinated with the HPV vaccine by the age of 15, 70% of women screened using a high-performance test by the age of 35, and again by the age of 45, 90% of women with pre-cancer treated and 90% of women with invasive cancer managed” [5, 6]. According to this strategy, “all countries must reach and maintain an incidence rate of below 4 per 100 000 women in order to eliminate cervical cancer” [5, 6]. In Nigeria however, the age standardized rates for cervical cancer is much higher, at 36.0 per 100,000 [7] and like several developing countries, uptake of cervical cancer screening is low [8–13] and many cases of cervical cancer present late, with attendant complications and mortality [14]. In Nigeria, about 12,075 new cases of cervical cancer are diagnosed annually and about 7,968 deaths from cervical cancer occur annually [15]. It is projected that by the year 2025, cervical cancer deaths in Nigeria would rise by 63% and 50% for women aged ≤ 65 and > 65 years respectively [16].

In Nigeria, the cervical cancer control program is not well developed. Opportunistic screening is being practiced in hospitals when women present with gynecological complaints. Cervical cancer screening is available in government tertiary hospitals, which are only a few in each State of the country. A few government owned secondary health facilities can provide cervical cancer screening. Some private hospitals and diagnostic laboratories also provide cervical cancer screening services. Government and Non-governmental organizations infrequently organize cervical cancer screening outreaches in communities and sometimes in slum areas.

Cervical cancer disproportionately affects women of low socioeconomic status. Poverty along with other socio-cultural practices such as early marriage and high parity have been identified as factors that increase women’s vulnerability to cervical cancer [17]. Women residing in urban slums who often have low levels of education and income are more likely to have less awareness of cervical cancer and its prevention which in turn may lead to inadequate screening [17]. Women in low resource settings also often have poor access to healthcare [18].

Though many intervention studies carried out to improve cervical cancer screening have shown increased uptake rates [19–21], some did not achieve their aim. [22–24] These studies were only able to improve knowledge of cervical cancer screening, but this did not translate to improved practice [22–24]. Community-based interventions to improve uptake of cervical cancer screening will benefit from initial situation analysis to assess opportunities and threats to a successful intervention. Understanding the perspectives of the community members themselves about screening for cancer of the cervix would be a good place to start as this forms an important aspect of community participation.

Community participation refers to the involvement of people in a community in projects and programs to solve their problems [25]. The community can participate during the needs assessment, planning, mobilizing, training, and implementation [25]. Community participation in the form of community conversations is shown to empower communities by allowing them to identify challenges and ways of solving them [26]. Hence in this study, FGDs were carried out in two slum communities in Lagos Nigeria to explore women’s knowledge of cervical cancer, barriers, and recommendations for screening, to guide a cervical cancer screening intervention. Findings from this study will be important for policymakers, and for designing intervention programs to increase uptake of cervical cancer screening especially among women in low resource settings.

## Methods

### Study setting

About 10% of Nigeria’s population is resident in Lagos State, with a population density of 5,926 persons per sq. km [27]. Lagos has 192 identified slum communities. Pap smear services are only available in a few public healthcare facilities in Lagos mostly among tertiary hospitals. Some secondary and tertiary level private hospitals and several diagnostic centres across the state also provide pap smear services.

The study was carried out in two slum communities in Lagos - Ago-Egun Bariga community and Otto- Ilogbo extension community. Both communities are characterized by poor housing conditions and a lack of basic social amenities like wholesome pipe-borne water, good drainage, and adequate sanitation and women who reside in both communities are mostly traders and have low incomes.

### Study design

This qualitative study was conducted as part of a larger quasi-experimental study that assessed the effect of a social marketing intervention on the knowledge, attitude, and uptake of pap smears among women residing in slums [28]. The intervention was a Pap smear screening program that was designed using the concept of social marketing. The 4Ps of the social marketing mix - Product (Pap smear procedure), Place (venue for the pap smear services), Price (the cost of the test) and the Promotion (advertising done) were taken into consideration in the intervention [28]. The FGDs were conducted before the social marketing intervention and were meant to guide all aspects of the intervention, including the marketing mix.

### Selection of participants

Two slums were selected from all the identified slums in Lagos. In each slum, participants were purposively selected. Women were approached face to face and had a screening interview to determine eligibility. The eligibility criteria were women aged 21–65 years who had resided in one of the two selected slums for at least one year and who were married/cohabiting or were sexually active. Forty – five eligible women were recruited, out of which 35 (77.7%) turned up for the FGDs.

### Data collection

Two focus group discussions (FGDs) were carried out in each community making four in total. The FGDs were conducted in a neutral location in each community, that guaranteed privacy and confidentiality. Groups of 8–10 women were used per FGD and a total of 35 women took part in the FGDs i.e. 17 women in Ago-Egun Bariga and 18 women in Otto-Ilogbo extension. In each community, one FGD was conducted among younger women aged 21–40 years, while the other was conducted among older women aged 41–65 years.

An FGD topic guide was developed based on previous literature and was pretested among a similar population. The FGD guide assessed the knowledge of cervical cancer and its prevention, perceived barriers to the implementation of a cervical screening program in their community, recommendations to overcome the barriers, the preferred location for the screening test, the preferred cost of the test, the best ways to publicize the program, and the measures to be taken to improve the way women perceive their susceptibility to cervical cancer (see Additional file 1). A short questionnaire was used to collect demographic information from the participants.

Two days before each FGD, participants were sent SMS reminders about the venue and time of the FGD and were visited at home by a community mobilizer. The FGDs took place within each community. Each FGD was moderated by the researcher who is female and a medical doctor with specialty in Public health. Trained female research assistants with a minimum of ordinary level diploma qualification served as note-takers and timekeepers. The FGDs were held in the local Yoruba and Egun languages and were audio-recorded, while a note-taker took notes. Informed consent was obtained from the participants.

The discussion began with greetings, the introduction of the researcher and research assistants, and participants were informed about the purpose of the FGDs. Following this, the researcher asked open-ended questions after which participants were encouraged to give their own opinion on each question raised. Prompts, probes and follow-up questions were used when necessary to encourage further discussion until there were no further discussions on that subject. Because most of the respondents did not have good knowledge of cervical cancer and cervical cancer screening, after eliciting responses on knowledge of cervical cancer and screening, a brief talk was given to explain what cervical cancer is, its symptoms and risk factors, its severity and how the disease can be prevented. The principal investigator thereafter explained that a cervical cancer screening program was in the planning stages for women in the communities and their input would be beneficial for planning. Further questions were then asked.

At the end of each session, the researcher summarized the proceedings and gave de-briefs to participants. Each session lasted about one hour. Unique identifiers were used for field notes, audiotapes and the questionnaires instead of participant names. Audiotaped recordings were transcribed verbatim in the local languages and then translated to the English language Data coding was done by the principal researcher and reviewed by a co-researcher to ensure rigor and trustworthiness.

Data were analyzed by using thematic analysis. A combination of inductive and deductive approach was used in coding. The process of analysis involved familiarization with the data, generation of initial codes, searching for themes, reviewing themes, defining and naming themes, and then writing the report.

## Results

### Themes and sub-themes

The results of the FGD provide information on women’s knowledge of cervical cancer, barriers and recommendations for screening, These results are presented in 4 broad themes: (i) Knowledge of cervical cancer (ii) Acceptability of a cervical cancer screening program. Iii) Barriers to a cervical screening program iv) Recommendations for a successful screening program (Table 1).

**Table 1:** Themes and Sub-themes

Themes	Sub - themes
**Knowledge of Cervical Cancer**	1. Awareness of cervical cancer 2. Symptoms, risk factors, and prevention of cervical cancer.
**Acceptability of the cervical cancer screening program.**	
**Barriers to a cervical screening program**	
**Recommendations for a successful screening program**	1. What the program can do to overcome the identified barriers 2. The Preferred Place for the test 3. The Preferred cost of the test 4. Best ways to publicize the program 5. Measures that can be put in place to increase perceived susceptibility to cervical cancer. 6. Ways to make cervical cancer screening sustainable in the community.

### Participant description

The participants demographic characteristics are shown (Table 2).

**Table 2:** Participant demographic characteristics

Variable	Frequency(n=35)
**Community**	
Ago-Egun Bariga (Slum 1)	17
Otto-Ilogbo extension (Slum 2)	18
**Age group**	
21 – 40 years	17
41 – 65 years	18
**Marital Status**	
Single	1
Married	34
**Highest Level of Education**	
No formal education	17
Primary education	4
Secondary education	11
Post-secondary	3
**Religion**	
Christianity	32
Islam	3
**Occupation**	
Unemployed	1
Traders	29
Tailors	2
Hairdressers	2
Teacher	1
**Monthly Income**	
N1000 ($2.40) < N10,000 ($24.39)	19
N10,000 ($24.39) < N20,000($48.78)	10
N20,000($48.78) – N30,000($73.17)	6
Average monthly income	N14,647 ($35.72)

1 $ = N410 (exchange rate at the time of the study)

### Knowledge of cervical cancer

#### Awareness of cervical cancer

Most of the women had never heard about cervical cancer. Only one woman had heard of cervical cancer. However, many were aware of breast cancer.

*‘I have not heard about it.’* was said by a 25-year-old woman, shaking her head [Slum 1, age 25].

*‘I have heard it affects people in the breast but not that it affects the mouth of the womb’* was said by a 50year old woman [Slum 2, age 50].

*‘Me, I have heard of it. They came to give us a talk on it in church when we were doing a women’s conference last year. The doctor that was invited to educate us told us about it*, was said by a 55year old teacher [Slum 1, age 55].

#### Symptoms, risk factors, and prevention of cervical cancer

None of the participants knew the symptoms or risk factors of cervical cancer. Some respondents had misconceptions of the risk factors of cervical cancer. Some participants were not clear if cervical cancer could be prevented or not, while others believed it could not be prevented. Only one respondent thought the disease could be prevented from frequent hospital check-ups. None was aware of the Pap smear test. When asked their opinions on cervical cancer, many of the women affirmed that all cancers are severe.

*‘I don’t know the symptom I can mention, but I know it is very dangerous.’*[Slum 2, age 42].

*‘Me, I think maybe dirt, or maybe when one does not take proper care of one’s self.’* [Slum 1, age 26].

*‘It can be prevented if a person sees her doctor frequently. If she does not wait too long to see her doctor.’* [Slum 2, age 55].

#### Acceptability of the cervical cancer screening program

Participants’ opined that the program will be well accepted in the community. Most discussants believed the program would be beneficial and would be well accepted especially if not expensive.

*‘Many people will come. Many many many people. So far it is for our own good*’ was said by a woman from the Ago-Egun Bariga community. [Slum 1, age 46]

*‘I feel as young as I am that such a test is for our benefit and so that we can live long lives. If it is not something that they will ask us to bring billions if it is what we can afford because money is what is important. Or if well-meaning Nigerians can help, I feel it is of benefit and will make our lives long.’* said a 26-year-old single lady from the Otto-Ilogbo extension community. [Slum 2, age 26]

#### Barriers to a cervical screening program

The respondents generally thought the pap smear test was good. They however expressed concerns about the cost of the test and the sex of the provider, performing the test. Most discussants preferred female providers while others were indifferent. Some participants stated that they will require the consent of their husbands to undergo screening and a few had superstitious beliefs.

*‘It is good. We are only concerned about the cost. If it is too expensive, some of us will not do it.’* said a 34-year-old trader. [Slum 1, age 34]

*‘The test is good but it is a woman that should treat a woman. A man cannot treat a woman. If it is a man that will perform the test, I will not do it, but if it is a woman, I will.’* said a 37-year-old woman. [Slum 2, age 37]

*‘If they don’t want to do it, the reason is money. They cant bring a good thing and women will say they will not partake of it.’* [Slum 1, age 52].

*‘We Egun, we have a taboo that says; except a woman is in labour or is having intercourse with her husband, she can’t be exposed. As you have come to do this test for us, we can go back home and tell our husbands. If my husband agrees, I am ready to do the test.’* [Slum 1, age 34].

*‘Whether it is a man or a woman, as far as it is for our protection. You can’t be having a child now, and they say it’s a man that will take the delivery, then the woman will close up her legs. Whether it is a man or woman, any doctor that is available is for our good.’* [Slum 1, age 29].

*‘This test you want to do for us, don’t make it inconvenient for us because we learned that you will collect money. The way we are, we are paupers, poor people. If the money is too much, many people will run away. Don’t let the price be inconvenient. Some people have not eaten breakfast and don’t know what their child will eat. If the price is high, such a person will run away.’* [Slum 2, age 21].

*‘Some people are afraid that maybe they want to take something from their body or turn around their womb so they can no longer have children, or it can make them begin to have heavy menstrual flow. Some people say they want to take our blood away.’* [Slum 1, age 54].

*‘Some people are afraid of the people coming. Are they mermaids?’* [Slum 1, age 65].

### Recommendations for a successful screening program

#### What the program can do to overcome the identified barriers

The respondents proffered some solutions to the identified barriers which include; reducing the cost of the screening test or providing it free of charge, having people that speak the local language in our team and using only females as health care providers for this purpose.

*‘If they bring the test, it is good. But if you say you want to bring this test, whether it is free, or at a cost, I don’t know. But if it is not free, please let the cost be little. It is good, bring it.’* [Slum 1, age 26].

*‘What you can do is that when you want to do the test, bring people along, like people that understand the language, so that they will explain to them, this is what you want to do, It is not that they want to do anything bad with you.’* [Slum 2, age 45].

*‘Bring female providers and they will allow. We have told you, that with the female providers, we are ready to do the test.’* [Slum 1, age 30].

#### The preferred place for the test

The majority of the discussants wanted the test to be carried out in their community. They also wanted a private place. A few thought that the test could be carried out either in a nearby PHC or within the community. One woman was willing to do the test at any venue available.

*‘If you can bring your instruments, you can do it in the community. Find a place that is secure and private. You will see more people ready to do the test.’* was said by a woman from the Otto-Ilogbo extension community. [Slum 2, age 52]

*‘ Look for somewhere in Ago Egun (the community), you will see many people, not anywhere outside Ago, because I cannot leave my work.’* [Slum 1, age 39].

*‘If it is in this neighbourhood or even in the PHC, both are good. There is one PHC now nearby that is new, that one is also ok. And if it is this neighbourhood, that is also good. There is none that is not good among the two options.’* [Slum 2, age 34].

*‘Tell us where to go, even if it is at your place of work.’* [Slum 1, age 24].

#### The preferred cost of the test

In the Ago-Egun Bariga community, among the younger women, some were willing to pay N500 ($1) for the screening test and others said it should be free. Among the older women, some stated that they could pay N1,000 ($2), some N500($1), and others said it should be made free.

*‘These times we are are hard. We are not making enough sales. If it is free, we will get many people but if it is N500, that is still ok.’* Said a 33-year-old fish trader. [Slum 1, age 33]

*‘If you say we should insist on N500, people will drawback a lot. Don’t let us insist on N500’.* [Slum 1, age 29]

*‘N1000 is ok. Even a person that does not attend the seminar, if he hears that the money is a little bit down, they will come.’* Said a 55-year-old teacher. [Slum 1, age 55]

*‘I thought it will be free.’* Said a 55-year-old trader. [Slum 1, age 55]

*‘As I am, I fetch water to sell. Do you understand? I was fetching water when they said you were around. By God’s grace, God will help us pay the bill. Let us leave it as N500’* said a 41-year-old woman from the Otto-Ilogbo community. [Slum 1, age 41]

*‘I myself, as I sit here, I have not had breakfast. If the children have not gone to the market and returned, I can’t see what to eat. So if the money is little, we will do it but if it is more than what we can afford, there is nothing we can do. If it is N500 it is still ok but not if it is more than that.’* [Slum 2, age 41].

*‘You see, this place is a slum, dump yard. There is no money. There are many people here that are suffering. There is no money. About paying money, that is not possible. Even when you are coming, come with food and water. What help you can do for us is what you should do. You don’t know how God will reward you, but just help us.‘* [Slum 2, age 22].

#### Best ways to publicize the program

Respondents suggested the use of SMS, phone calls, and health talks to publicize the program. They also recommended that a community member could move around the community to mobilize people for the program using a megaphone.

*‘The same way you called us to talk to us, call them.’* [Slum 1, age 24].

*‘Use a megaphone 2 days before, the message will also spread by word of mouth’* [Slum 1, age 41].

*‘If you call us on phone we will come, if you use text messages we will come.’* [Slum 2, age 24].

### Measures that can be put in place to increase perceived susceptibility to cervical cancer

The respondents said that providing health education can help increase women’s perceived susceptibility to cervical cancer.

*‘If you call them together to talk to them, they will hear. But this time you came is a ‘hot time’ for many people. Choose a time when most people are less busy, maybe on a Sunday evening.* [Slum 1, age 46]

*‘Let them understand that every woman can have the disease. Explain to them the way you have explained to us that the disease starts gradually and progresses.’* [Slum 2, age 42].

*‘Give them a health talk but if they don’t listen, there is nothing you can do about it.’* [Slum 2, age 34].

### Ways to make cervical cancer screening sustainable in the community

Some of the respondents were of the opinion that after receiving health education, women who are convinced would continue to undergo the test, even after the program has ended. It was recommended by some participants that reminders be sent every three years to remind women to undergo cervical cancer screening. One respondent said the team should come back in three years to repeat the tests and another wanted our team to come and build a health facility in their community.

*‘If you explain to us well, people will go to the hospitals afterwards, to do the test.‘* [Slum 2, age 43].

*‘The step you can take is what you have taken already. As those of us here understand well, even if you go elsewhere, when that person is convinced, he will go for the test.’* [Slum 2, age 22].

*‘Let them have reminders in the next three years. Some will go and do the test.’* [Slum 2, age 24].

*‘The nurses that do the test for us, you can leave them here with us, so that they will be doing it for us every three years. We will be very happy.’* [Slum 1, age 60].

*‘Come and build your hospital here. We will then know that your hospital is here. When we leave home, we will be coming straight there.’* [Slum 1, age 54].

*‘Whether you build your hospital here or not, the person that knows well about the disease and does not want to die will go for the test.’* [Slum 1, age 26].

## Discussion

Awareness of cervical cancer was low in this study. Other studies carried out in slum communities have also reported very low awareness of cervical cancer [8, 10, 29–31]. The low socio-economic status and low level of education of these groups of women may explain the low awareness of cervical cancer seen in our study as some studies carried out among well educated Nigerian women have shown higher awareness [32, 33]. The low awareness of cervical cancer may also be related to the hidden anatomical location of the cervix, as it is away from view unlike the breast as many women were aware of cancer of the breast but had never heard of cancer of the cervix. Breast cancer is also the commonest cancer among women in Nigeria, hence they may have seen or heard about someone with the disease.

Most of the discussants did not know the symptoms and risk factors of cervical cancer, and the majority did not know if cervical cancer could be prevented or if there were any screening tests for cervical cancer. Similarly, in a qualitative study involving women from two cities in Serbia, poor knowledge of cervical cancer screening was a predominant theme in all focus groups [34]. In contrast in a study in rural communities in Eastern Uganda, the majority of the respondents knew that cervical cancer could be prevented and most could correctly state at least one preventive measure of the disease [35]. The higher knowledge of prevention of cervical cancer in the Ugandan study may be as a result of more media campaigns on cervical cancer in Uganda, as most respondents in that study had got their information from radio [35].

Most of the participants believed the program would be well accepted by community members especially if not expensive or provided free. This view was however expressed after the women had been briefed about cervical cancer, its symptoms, risk factors, complications, and prevention. Barriers to screening highlighted by the discussants in our study include; the sex of the person performing the test, financial constraints, requiring consent from husbands, and superstitious beliefs. Due to cultural and religious beliefs, it is not uncommon for women to shy away from vaginal examinations by male health workers especially when it is not a medical emergency or not during the process of childbirth. A similar finding was reported by Modibbo et al. in a study conducted in two hospitals, in the southwest and northcentral regions of Nigeria [36]. In a study among Latino women living in California, most women showed a preference for female health workers [37]. A study carried out in Eastern Uganda reported that many women felt uncomfortable undressing before male health workers [38].

In our study, several women wanted the test to be carried out free of charge. Some were willing to pay N500($1), an amount of money that would buy one loaf of bread, and a few were willing to pay N1000($2). The group of women interviewed was of low socioeconomic status. Most of them were small-scale traders and earned less than the monthly Nigerian minimum wage, N18,000 at the time ($44). It is thus not surprising that many were unwilling to pay for the test. Similarly, in a study among uninsured women in Texas, the majority of respondents identified cost as a barrier to receiving a Pap test [39]. Low level of income was identified as a barrier to cervical cancer screening in a study among rural women in Ghana, as some respondents explained that poverty prevented them from going to screen [40].

Findings from our study show that some women will require the consent of their husbands to undergo cervical cancer screening. In the traditional African society, men assume the role of decision-makers even when it has to do with the health of their wives, hence our finding [41, 42]. This normative gender role could constitute a barrier to screening because some women may not want to undergo the hassle of seeking permission from their husbands. Furthermore, husbands may have a poor understanding of cervical cancer screening and demonstrate misconceptions, hence preventing their spouses from undergoing screening. Similar to our study, in a study in a rural community in Lagos Nigeria, spousal approval was needed for women to undertake cervical cancer screening [43]. Similar finding was reported in a rural district in Tanzania [44] and rural Ghana [40]. Educating men about the importance of cervical cancer screening may help improve uptake of screening among their spouses.

Some discussants raised concerns about rumors making rounds in the community that the team was going to perform diabolical rituals on them. It was thus suggested that involving people from the community who are well known and trusted by these women, and who speak the local language in the program will go a long way to allay their fears. This is a form of community participation, which has been proven to improve community ownership of health programs.

Cervical cancer screening outreaches are sometimes done in health facilities and can also be organized in communities. The majority of the discussants wanted the test to be carried out in a private location in their community. This may be because it makes the process very convenient, without the need to incur transportation costs and limits the time spent away from their work and families. The women may also feel more comfortable in a familiar environment. The participants may have preferred the community because they were asked where they want the tests to be carried out, and their options were not limited. In a scenario where the option of a community venue is not the case, they may have gone for the next closest option as a few women were of the opinion that the screening could be carried out in a nearby primary health centre. However, it can be said that the discussants wanted the tests to be carried out as close to them as possible.

Many studies among Nigerian women have reported low perceived susceptibility to cervical cancer [8, 31, 32, 45]. All sexually active women are at risk of developing cervical cancer hence the need for all sexually active women to have this understanding. A good intervention aimed at increasing uptake should strive at increasing women’s perceived susceptibility. In this study, when discussants were asked about measures that can increase women’s perceived susceptibility to cervical cancer, many said health education sessions will help. Some studies have also shown that health education can improve women’s attitudes towards cervical cancer. In a health education intervention study among rural women in Ogun State, Nigeria [46] and in a health education intervention study among teachers in Kebbi State Nigeria [22], attitude towards cervical cancer increased significantly after health education.

When asked about ways to make Pap smear testing sustainable in the community, **s**ome discussants thought that with health education, some women would continue to undergo Pap smear testing even after the program has ended, at locations were these are available e.g. hospitals. It was recommended by some participants that reminders be sent every three years to remind the women to go for Pap smear testing. Sending reminders has been shown to be effective in promoting Pap smear testing. In Manitoba, Canada [47], and Ontario, Canada [48], reminders were sent to women in form of invitation letters and this resulted in higher uptake of Pap smear compared to the women who were not sent reminders. One respondent wanted a repeat Pap smear screening program in three years while another wanted a health facility built in their community for that purpose. Although this may not be always feasible, it goes to show that the women would appreciate it if encountering geographical barriers while seeking care was not the case.

### Strengths and limitations

Many qualitative studies have identified deterrents to cervical cancer screening among different populations [36, 37, 49–52]. In our study, in addition to identifying barriers to screening, we elicited suggestions from women that could guide the design of a successful cervical cancer screening program and our findings have the potential to influence the implementation of similar programs, especially among women living in low resource settings. A limitation of this study is that participants in our study were limited to two slum communities in Lagos Nigeria and results may not be generalizable to different settings. In addition, two FGDs were conducted in each slum community to serve as basis for designing the social marketing intervention. Even though the decision to conduct four FGDs was not initially based on data saturation, a lot of valuable information can be obtained from our findings that can guide interventions. Additionally, after data familiarization with the audio recordings and transcripts, we noticed that by the third and fourth FGD, responses were similar among most participants and no new information were added.

## Conclusion

Knowledge of cervical cancer and cervical cancer screening was poor among the women. We identified barriers and recommendations for a successful cervical cancer screening program which included reducing the cost of the test or making it free, using a private venue within the community or a nearby PHC, using female providers, and publicizing the program with the use of SMS, phone calls, town crier, and health talks and having people that speak the local language as part of the team. Educating men about the importance of cervical cancer screening may also help improve uptake of screening among their spouses as women reported requiring consent from their spouses to undergo screening. Though not all programs may be able to address all the concerns and institute all the recommendations, it is, however, important that policymakers and programmers consider some, if not all these recommendations in designing effective cervical cancer screening interventions among women in low resource settings.

## Electronic supplementary material

Below is the link to the electronic supplementary material.

Focus group discussion topic guide.

## Data Availability

The data used and/or analysed during the current study available from the corresponding author on reasonable request.

## List of abbreviations

ASIR: Age-standardized incidence rate .
ASMR: Age-standardized mortality rate.
FGD: Focus group discussion
PHC: Primary health center.
SMS: Short message service.
