# Supplementary material for: Barriers and recommendations for a cervical cancer screening program among women in low-resource settings in Lagos Nigeria: a qualitative study
Source: BMC Public Health. 2022 Oct 12;22:1906. doi: 10.1186/s12889-022-14314-2 (PMC9560022; doi:10.1186/s12889-022-14314-2)
Supplement: Supplementary file 1 — Focus group discussion topic guide. [file 12889_2022_14314_MOESM1_ESM.docx]

**Focus group discussion topic guide**

1. ‘‘When you think about cervical cancer, what comes to mind?’’
2. ‘‘What do you think are the causes of cervical cancer?’’
3. ‘‘What do you think are the symptoms of cervical cancer?’’
4. ‘‘What do you feel about the severity of cervical cancer?’’
5. ‘‘Do you think cervical cancer can be prevented? please tell me more’’
6. ‘‘When you think about cervical cancer screening and pap smear, what comes to mind?’’
7. ‘‘Do you have concerns about undergoing a pap smear? Please explain.’’
8. ‘‘What is the situation in your community as pertains cervical cancer screening? Please help me understand.’’
9. ‘‘What do you feel about the acceptability of a cervical cancer screening programme in your community?’’
10. ‘‘Could you please explain the likely barriers women may encounter and concerns they might have in assessing pap smear services within your community?’’
11. ‘‘What measures do you think can be put in a place to improve pap smear testing in your community?’’
12. ‘‘Where would you like to have the pap smear?’’
13. ‘‘How much will you be willing to pay for the pap smear?’’
14. ‘‘In what ways do you think we can encourage women to undergo pap smear?’’
15. ‘‘What ways can we promote pap smear in the community?’’
16. ‘‘What ways can we improve women’s feeling of perceived susceptibility?’’
